# Interface Charge Induced Multifunctional Manipulations in Metals

**DOI:** 10.1002/advs.76628

**Published:** 2026-07-14

**Authors:** Qiang Cao, Yingli Li, Senmiao Liu, Zhen Han, Weixin Liu, Zhaohui Li, Yufeng Tian, Xinglong Ye, Shengshi Li, Qiang Li, Shishen Yan

**Affiliations:** ^1^ Spintronics Institute University of Jinan Jinan China; ^2^ College of Physics, Weihai Innovation Research Institute, Institute of Materials for Energy and Environment Qingdao University Qingdao China; ^3^ Department of Electrical and Computer Engineering National University of Singapore Singapore Singapore; ^4^ School of Physics, State Key Laboratory of Crystal Materials Shandong University Jinan China

**Keywords:** control of magnetism and resistivity, double interfaces, metal‐oxide composite heterojunction, space‐separated electrons and ions

## Abstract

Metals are indispensable in modern information technology due to their unique magnetic and electronic properties. However, achieving voltage control of these properties in common metals with large amplitude, reversibility, and durability remains a formidable challenge. Here, we demonstrate giant and reliable tunning of magnetic anisotropy, magnetization, and resistivity in Pt/Co bilayers through an interface‑charge mechanism. By integrating a TiO_2_/Pt/Co/TiO_2_ heterojunction as positive electrode in a lithium‐ion storage device, lithium‐ions migrate into the TiO_2_ layers during discharge, while electrons are simultaneously injected into the Pt/Co layer through external circuit. The space‐separated ions and elections accumulate at TiO_2_/Pt and Co/TiO_2_ interfaces, generating strong interfacial capacitor effect and high‑density electron accumulation in the metals. In high voltage range (3–2 V), the interfacial capacitance reorients Co anisotropy from perpendicular to in‐plane. Further voltage reduction to 0.8 V induces charge accumulation at both interfaces, resulting in 44% decrease in saturation magnetization of Co and 55% reduction in longitudinal resistance of Pt layer. These findings establish the interface‐charge as a robust and versatile platform for giant, reversible, and reliable voltage control in metals, opening new opportunities for spintronics, sensing, and neuromorphic computing.

## Graphical abstract

Voltage‑controlled interface charge enables giant, reversible modulation of magnetic anisotropy, magnetization, and resistivity in common metals. Space‑separated ions and electrons accumulate at interfaces, producing strong interfacial capacitor effect and high‑density electrons injection. This robust mechanism preserves layer integrity while offering reversibility and stability, opening pathways toward multifunctional devices in spintronics, sensing, and neuromorphic computing.

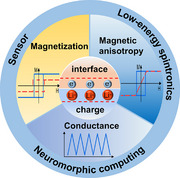

## 1 Introduction

Metals are vital for modern information technology because they provide exceptional electrical conductivity and magnetic functionality. Achieving voltage control of both properties in metals holds transformative potential for next‑generation computing [1, 2], data storage [3, 4], and communication technologies [5, 6]. Nevertheless, the short screening length in metals arising from their high carrier density, severely limits the effectiveness of conventional electric‐field modulation. Therefore, electrolyte gating has attracted lots of attention as a promising strategy to manipulate metallic properties through ion‑controlled or ion‑mediated electronic processes [7, 8]. The first major breakthrough in this field is the electric double layer (EDL) [9]. When a voltage is applied across an electrolyte and a metal, ions in the electrolyte rearrange to form a compact capacitor at the interface, thereby enhancing the local electric field at the metal surface. Notably, voltage only drives ions to the interface, while the carrier density inside the metal remains unchanged. As a result, the EDL alone cannot directly modulate magnetization or resistance, restricting its influence to surface‐level phenomena. To this end, subsequent strategies such as ion intercalation [10, 11] and redox reactions [12, 13, 14, 15] have been emerged, which enable penetration of ions into the lattice or chemical modification of the material, thereby achieving more effective control over electronic and magnetic properties [16, 17, 18, 19]. Despite notable progress, these electrochemical processes often suffer from prolonged operating time, elevated temperature, and material degradation over repeated cycles [12, 13, 20, 21, 22], which compromise both reversibility and long‐term reliability.

To overcome these limitations, we propose adopting the interface‐charge mechanism [23] to achieve voltage control of magnetic and electric properties in common metals with giant amplitude, reversibility, and durability. This strategy decouples ionic and electronic conducting pathways: ions are stored in an ion‐conducting layer under applied voltage, while electrons accumulate in an adjacent metallic film. The dual transport pathways of ions and electrons enable exceptionally high charge accumulation, with capacitance densities reaching ≈10^15^ cm^−2^, far exceeding the ≈10^13^ cm^−2^ limit of conventional EDLs [23]. Moreover, such spatial separation allows non‐faradaic modulation without subjecting the active layer to direct ionic infiltration, thereby preserving the integrity of the functional layer and offering relatively faster switching speeds and improved cyclic stability compared with conventional electrochemical processes. Theoretically, microsecond‑to‑nanosecond operation has been predicted [24]. Furthermore, unlike traditional electrolyte gating, the flexible spatial distribution at ion‐conductor/metal solid interfaces allows vertical stacking of multiple units with diverse metallic compositions, thereby offering a scalable route toward multifunctional device architectures by layer‑specific tuning of charge and spin degrees of freedom.

In this study, we demonstrate giant and reliable non‐faradaic tuning of magnetic anisotropy, magnetization, and electrical resistivity in Pt/Co bilayer through interface‐charge mechanism. By integrating the TiO_2_/Pt/Co/TiO_2_ heterojunction as a positive electrode in a lithium‐ion storage device, interface‐charge is formed during discharge at both interfaces, Co/TiO_2_ and TiO_2_/Pt. At the Co/TiO_2_ interface, interface‐charge generates an interfacial capacitor within high voltage range and high‐density electron accumulation in Co within low voltage range, which not only reorients the Co magnetic anisotropy from perpendicular to in‑plane but also induces a giant saturation magnetization (*M*_s_) reduction in Co by up to 44%. Meanwhile, the interface‐charge at the TiO_2_/Pt interface produces a substantial resistance decreases in Pt layer by up to 55%. All these manipulations are fully reversible and exhibit excellent durability, establishing the interface‐charge as a powerful and versatile platform for multifunctional control in metallic systems, thereby laying the foundation for multifunctional devices in spintronics, sensors, and neuromorphic computing.

## 2 Results

### 2.1 Space‐Separated Ions and Electrons at Double Interfaces

**FIGURE 1:**
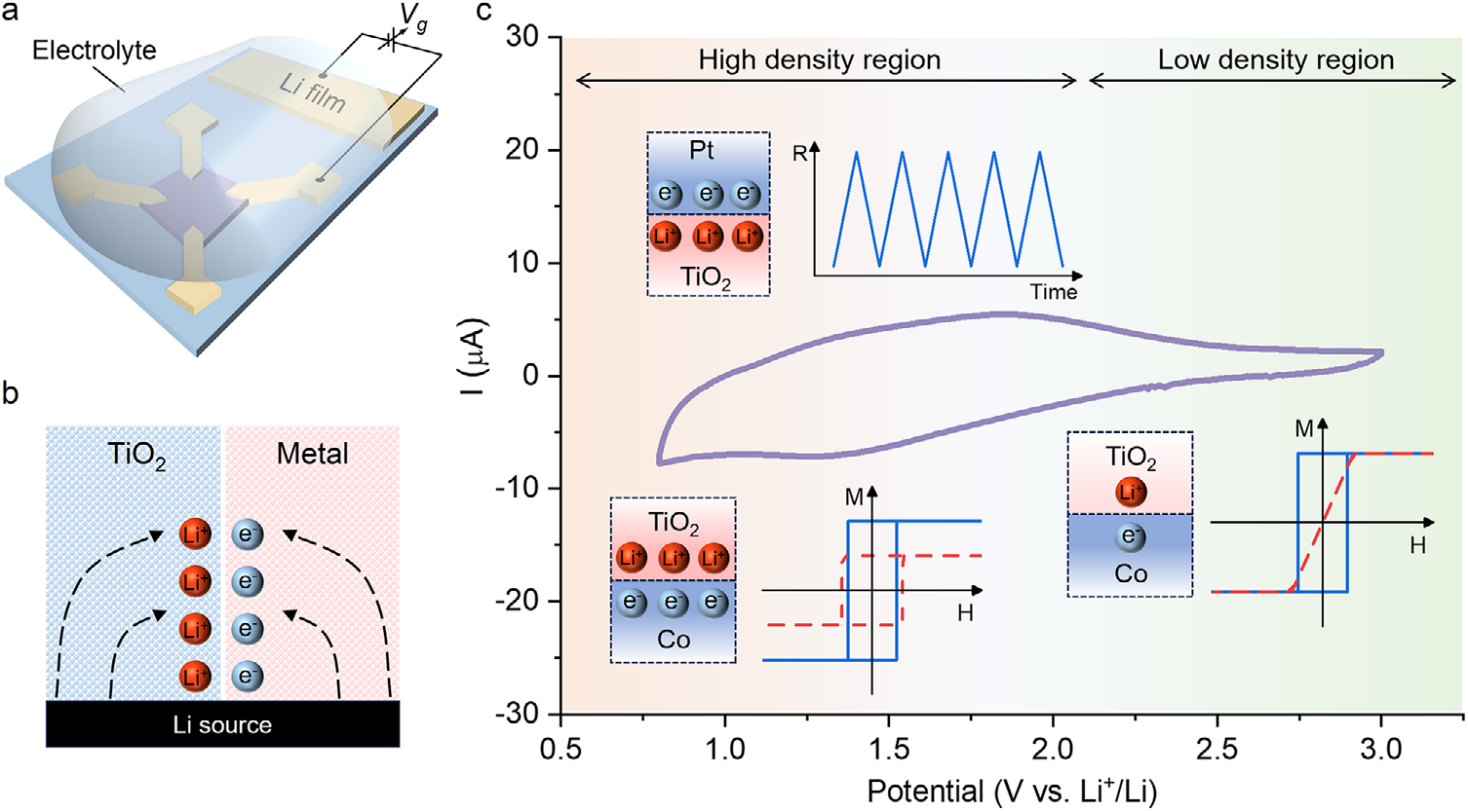
(a) Schematic illustration of the device architecture and Van der Pauw measurement configuration. (b) Schematic of the space separated ions and electrons storage mechanism. (c) CV profile obtained over the voltage range of 3 to 0.8 V, revealing two distinct regions: a low charge density region in high voltage range and a high charge density region in low voltage range. In the low‐density region, spatially separated ions and electrons induce an interfacial capacitance, affecting the magnetic anisotropy of Co layer. In the high‐density region, more electrons accumulated in the metals causes the reductions in *M*_s_ of Co and resistance of Pt.

Figure 1a illustrates the schematic of our device, comprising a TiO_2_ (3 nm)/Pt (2 nm)/Co (1 nm)/TiO_2_ (3 nm) heterojunction as a positive electrode, with a lithium film as the negative electrode and LiPF_6_ as the electrolyte. In the positive electrode, TiO_2_ layers function as an ion conductor, selectively accommodating Li^+^ ions while excluding electrons [25]. Conversely, electron accumulation occurs exclusively within the metallic Pt/Co layer. Upon application of a positive voltage V_g_ (discharge), Li^+^ ions and electrons are injected and spatially separated in the TiO_2_ and Co/Pt layers, respectively, as depicted in Figure 1b. The cyclic voltammogram (CV) over the full voltage range (Figure 1c) reveals no distinct redox peaks, indicating the absence of faradaic reactions. Complementary galvanostatic charge‐discharge curves (Figure S1) exhibit capacitive‐like behavior without discernible electrochemical plateaus [26]. Moreover, X‐ray photoelectron spectroscopy (XPS) of Co 2p and Pt 4f confirms the preservation of metallic states across the entire voltage range (Figure S2), consistent with the above results and supporting the conclusion that the modulation arises from non‑faradaic interfacial charge processes rather than bulk redox activity.

The CV profile reveals two distinct regions of charge transfer process: the low charge density region at high voltage, and the high charge density region at low voltage. In the first discharge stage (3–2 V), Li^+^ ions and electrons are separately injected into the TiO_2_ and metal layers at low density. Their spatial separation induces interfacial capacitance, which affects the magnetic anisotropy of Co layer. As the discharging continues (2–0.8 V), more ions and electrons are injected and accumulated at the TiO_2_/metal interfaces. In this region, the CV curve becomes nearly rectangular, characteristic of capacitive behavior. The high‐density electron accumulation within the metal layers enables effective modulation of magnetization and conductance. To elucidate the multifunctionality enabled by the interface‐charge, we systematically dissect the three key modulations: magnetic anisotropy, saturation magnetization, and longitudinal resistance, in the following sections.

### 2.2 Magnetic Anisotropy Modulation within the Low‐Density Region

**FIGURE 2:**
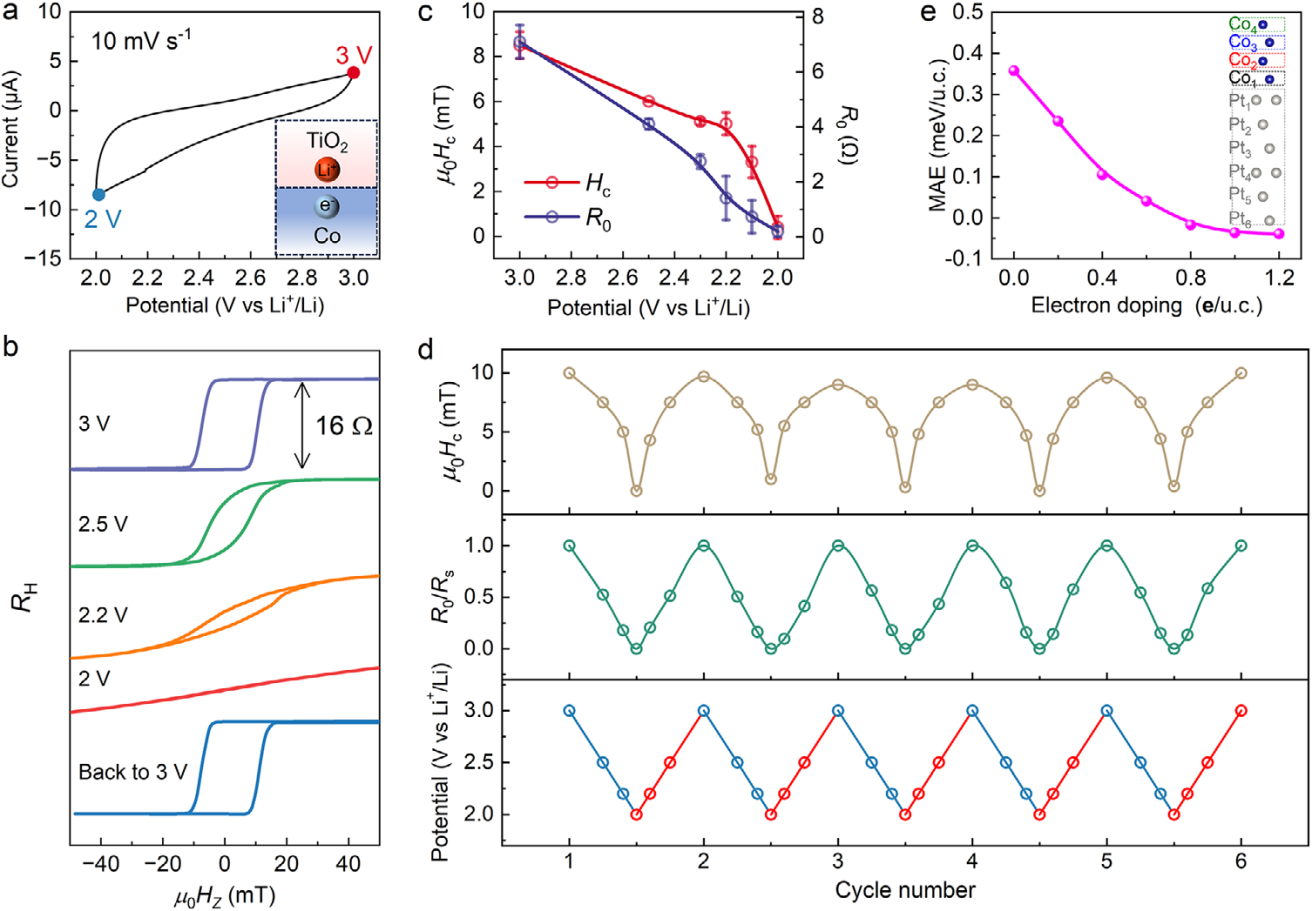
(a) CV curve recorded from 3.0 to 2.0 V at a scan rate of 10 mV s^−1^. Insert illustrates the interfacial capacitor formed by space separated ions and electrons. (b) Evolution of anomalous Hall resistance (*R*_H_) loops under sequential voltage steps from 3.0 V to 2.0 V and back to 3.0 V. To achieve a stable state at each voltage stage, the voltage is held for 10 minutes prior to *R*_H_ loops. (c) Voltage‐dependent variation of coercivity (μ_0_ *H*_c_) and remanence (*R*_0_). (d) Reversible and dynamic modulation of μ_0_ *H*_c_ and *R*_0_ derived from consecutive *R*_H_ measurements. A 10‐minute voltage hold is applied before each data acquisition to stabilize the magnetic states. (e) Dependence of the MAE of the CoPt multilayer on electron doping concentration. The inset shows the structural illustration of the CoPt multilayer.

The CV curve from 3 to 2 V is shown in Figure 2a, in which no discernible redox peaks are observed. Complementary CV measurements at varying scan rates reveal a near‐surface capacitor activities (Figure S3). In this region, ion‐electron injection and spatial separation generates an interfacial capacitor, significantly altering the magnetic anisotropy of Co layer. As shown in Figure 2b, the anomalous Hall resistance (*R*_H_) loops evolve with voltage, revealing a progressive reorientation of the Co magnetic easy axis from out‐of‐plane to in‐plane. Besides, it is fully reversible upon returning the voltage to 3.0 V. Figure 2c quantifies the voltage‐dependent changes in coercivity (μ_0_ *H*_c_) and remanence (*R*_0_), both of which decrease steadily as the voltage is reduced to 2.0 V. Figure 2d further demonstrates the dynamic and reversible modulation of μ_0_ *H*_c_ and *R*_0_ through repeated voltage sweeps between 3.0 V and 2.0 V, confirming stable and repeatable switching of magnetic anisotropy. To evaluate the strength of magnetoelectric coupling, the change in magnetic anisotropy energy (ΔK_i_) driven by applied voltage reaches 0.11 mJ V^−1^m^−2^ (Figure S4).

Control experiments highlight the critical role of the interface charge at Co/TiO_2_ interface in enabling anisotropy modulation. As shown in Figure S5, anisotropy remains tunable when the top TiO_2_ is retained. In contrast, replacing top TiO_2_ with MgO or Pt, materials that are incapable of Li^+^ ions storage, eliminates the modulation effect. This indicates that the interface‐charge at TiO_2_/Co interface is essential to switch magnetic anisotropy. Moreover, we examine Si/Pt/Co(x)/TiO_2_ single‑interface structures with varying Co thicknesses (Figure S6), confirming that observed anisotropy modulation originates from the Co/TiO_2_ interface. Furthermore, we perform first‐principles calculations to elucidate the magnetic anisotropy modulations. Under an applied voltage, Li^+^ ions intercalate into TiO_2_ while electrons are concurrently injected into the CoPt system. To simulate this process, we use an electron‐doped CoPt multilayer for our investigation. The magnetic anisotropy energy (MAE) of the CoPt multilayer is defined as MAE = *E*_100_ − *E*_001,_ where *E*_100_ and *E*_001_ denote the total energies with magnetization constrained along the in‐plane [100] and out‐of‐plane [001] directions, respectively. A positive MAE indicates a preference for perpendicular magnetic anisotropy (PMA), while a negative value corresponds to in‐plane magnetic anisotropy (IMA). Before electron doping, the CoPt multilayer exhibits PMA, as shown in Figure 2e. Atom‐resolved MAE analysis reveals that Co atoms yield positive contributions to the MAE, while Pt atoms collectively yield negative contributions; the dominance of the Co contribution over that of Pt results in PMA in the CoPt system (Figure S7). Upon electron doping of CoPt, a large portion of the doped electrons localizes on the surface Co atoms (Figure S8), consistent with the experimentally expected electron injection at the Co surface induced by interface charge effect. Interestingly, with increasing electron doping concentration beyond 0.8 **e**/u.c., the MAE of CoPt switches from positive to negative (Figure 2e), indicating a transition of the easy magnetization direction from out‐of‐plane to in‐plane. This phenomenon arises from a significant decrease in the MAE contribution from the Co layers, particularly from the Co_2_ and Co_3_ atoms, with the Co_3_ contribution becoming negative above 0.8 **e**/u.c. (Figure S9). To elucidate the MAE changes of the Co_2_ and Co_3_ atoms, we further analyze their orbital‐resolved MAE under both undoped and 1.2 **e**/u.c. doped conditions. In the undoped state, hybridizations between *d*_x2−y2_ and *d*_xy_ orbitals, as well as between *d*_xz_ and *d*_yz_ orbitals in the Co_2_ and Co_3_ atoms contribute to PMA, whereas the hybridization between the *d*_z2_ and *d*_yz_ orbitals gives rise to IMA (Figure S10a,b). Since the PMA contribution dominates, they both exhibit an out‐of‐plane easy magnetization axis. In the doped state, however, the positive MAE contribution from the hybridization between *d*_xz_ and *d*_yz_ orbitals decreases dramatically, reducing the MAE of the Co_2_ and reversing the sign of Co_3_ (Figure S10c,d).

The projected density of states (PDOS) of Co atoms for the undoped and doped systems is further calculated to clarify this change (Figure S11). Near the Fermi level, the electronic states are predominantly spin‐down. According to second‐order perturbation theory [27], MAE originates from interaction between occupied and unoccupied spin‐down *d* orbitals (i.e., *d* ^o−^ and *d* ^u−^). For Co_2_ and Co_3_, the interaction between occupied spin‐down *d*_yz_ orbitals (*d* ^o−^_yz_) and unoccupied spin‐down *d*_xz_ orbitals (*d* ^u−^_xz_) contributes to PMA (Figure S11b,c). Upon electron doping, the *d*_xz_ orbitals become occupied. In this scenario, the PMA comes from the interaction between occupied spin‐down *d*_xz_ orbitals (*d* ^o−^_xz_) and unoccupied spin‐down *d*_yz_ orbitals (*d* ^u−^_yz_) (Figure S11f,g). Since the DOS of *d* ^u−^_yz_ is much smaller, the PMA contribution weakens accordingly. For Co_1_ and Co_4_, the MAE shows only minor changes (Figure S9) with electron doping. Their electronic states near the Fermi level are mainly contributed by the *d*_xy_ orbital (Figure S11). The electron‑filling‑induced change in the *d*_xy_ orbital distribution hardly alters the orbital interactions and thus has little effect on the MAE. In brief, electron injection modifies the orbital distribution and hybridization of the Co layer, thereby switching the easy magnetization direction of the CoPt from out‐of‐plane to in‐plane.

### 2.3 Saturation Magnetization (*M*_s_) Modulations within the High‐Density Region

**FIGURE 3:**
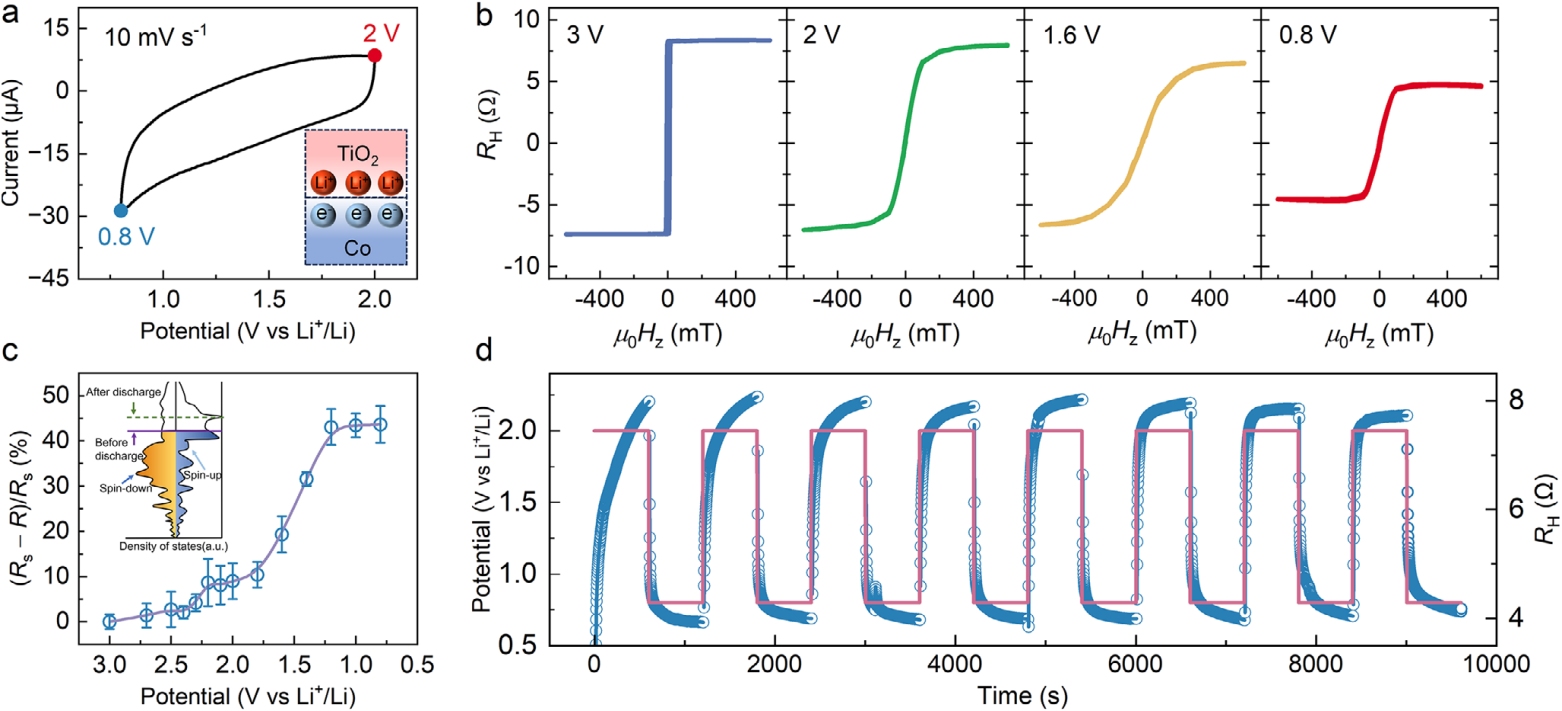
(a) CV recorded from 2 to 0.8 V at a scan rate of 10 mV s^−1^. Insert illustrates the ions and electrons accumulation at the TiO_2_/Co interface. (b) *R*_H_ loops evolution under various voltages from 3 to 0.8 V. (c) The corresponding *R*_H_ variation with voltage_._ Inset: schematic of spin‐polarized density of states in ferromagnetic metal. The electron injection elevates the Fermi level. (d) Temporal evolution of *R*_H_ under external field of 800 mT by repeatedly applying 2 and 0.8 V voltage. Each voltage is set to 600 s.

As the discharging process continues, more Li^+^ ions and electrons are accumulated at the Co/TiO_2_ interface. The accumulated electrons in Co significantly influences the *M*_s_. Figure 3a shows the CV recorded from 2.0 to 0.8 V. The rectangular shape of the CV curve is characteristic of capacitance. Additional CV measurements at varying scan rates confirm the capacitive nature of this voltage region (Figure S12). The huge electrons accumulation in the Co layer results in pronounced *R*_H_ variation related to the *M*_s_. Figure 3b displays the evolution of *R*_H_ loops as the voltage decreases from 3.0 to 0.8 V. The *R*_H_ values, drop by up to 44%, from 7.8 Ω to 4.4 Ω. As shown in Figure 3c, this *R*_H_ change differently in two regions. In the low‐density region between 3 and 2 V, the variation is minimal due to moderate electron injection; below 2.0 V, huge electron injection and accumulation results in a sharp decline in *R*_H_. This behavior is consistent with the spin‐polarized density of states in 3*d* ferromagnetic metals (Figure 3c, inset), where spin‐minority states dominate near the Fermi level. Electron injection preferentially fills the spin‐minority bands, reducing the net spin polarization and thereby decreasing the *M*_s_ [28, 29]. Moreover, by monitoring the changes in longitudinal resistance during this process, we find that *R*_H_ continues to exhibit significant modulation even when longitudinal resistance remains unchanged under applied voltage (as demonstrated in the next section). This rules out impurity scattering contributions such as side‐jump and skew scattering, indicating that the change in *R*_H_ is driven by variations in *M*_s_ following *R*_H_ = *R*_s_ *M*_s_, where *R*_s_ is the anomalous Hall coefficient [30]. Because longitudinal resistance is invariant, any independent change in *R*_s_ would necessarily perturb the longitudinal transport channels in ultrathin transition metal films, since they are tightly bounded. Thus, the observed 44% reduction in the saturated Hall signal reflects a genuine suppression of the out‐of‐plane magnetization rather than a trivial rescaling of *R*_s_.

Figure 3d illustrates the continuous switching of *R*_H_ under a saturation magnetic field of 800 mT. 2 V‐voltage pulses rapidly enhance *R*_H_, followed by gradual stabilization at elevated levels. Conversely, 0.8 V‐voltage pulses induce a swift reduction in *R*_H_, settling into a low stable state. This reliable and reversible modulation is attributed to the capacitive nature of interface‐charge, which enables consistent electron injection and extraction. Notably, the relatively slow modulation here was chosen to achieve stable states and to clearly demonstrate both reversibility and large modulation magnitude. Faster switching speeds are achievable, as shown in Figure S13. The *R*_H_ can be manipulated in a reversible manner when applying 10 pulses with a width of 50 ms, highlighting the capability of the system to operate on shorter timescales under optimized pulse conditions. To confirm the *R*_H_ control originates from the Co/TiO_2_ interface, control experiments are conducted (Figure S14). When the top TiO_2_ layer is retained, *R*_H_ remain tunable. However, replacing the top TiO_2_ with Pt eliminates modulation effects, as Pt cannot accommodate Li^+^ ions. In this case, electrons accumulation forms on the Pt surface rather than at the Co interface, leaving Co magnetism unaffected.

### 2.4 Resistance Modulations within the High‐Density Region

**FIGURE 4:**
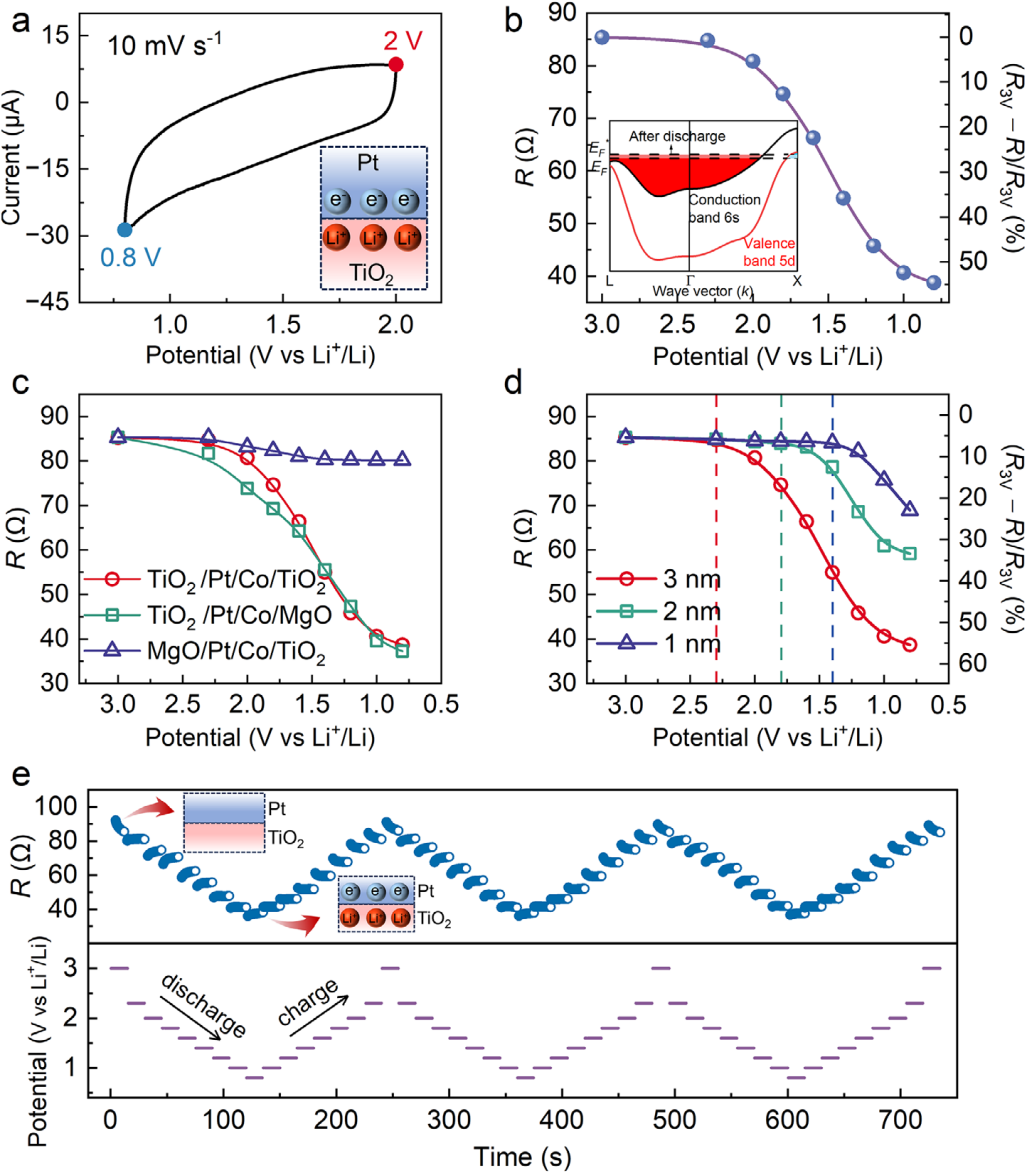
(a) CV recorded from 2 to 0.8 V and charge accumulation at the TiO_2_/Pt interface. (b) The resistance reduction with various voltages. The inset illustrates Fermi level (*E*_F_) elevation by electron accumulation in a simplified band structure of Pt, where the Fermi surface of pristine Pt is composed of 5*d* and 6*s* states at χ and Γ points of the Brillouin zone, respectively. (c) The dependence of resistance with voltage for three different structures: TiO_2_ (3)/Pt (1)/Co (0.5)/TiO_2_ (3), TiO_2_ (3)/Pt (1)/Co (0.5)/MgO (3), and MgO (3)/Pt (1)/Co (0.5)/TiO_2_ (3). The numbers in brackets represent nanometers. (d) The resistance variation of TiO_2_ (t)/Pt (1)/Co (0.5)/TiO_2_ (3) multilayer as a function of voltage, where *t* = 3, 2, and 1 nm. (e) Lithiation and de‐lithiation induced resistance evolution over time by applying a series of continuous ramped pulse voltages with 15s pulse width.

It is worth emphasizing that a key advantage of interface‐charge lies in its universality, enabling the formation of diverse interfacial configurations well beyond conventional electrolyte boundaries. This universality allows flexible integration of multiple interfaces within a single device, thereby facilitating compatible modulation of various physical properties. In our TiO_2_/Pt/Co/TiO_2_ heterojunctions, during discharge to 0.8 V, interface‐charge formation not only at the Co/TiO_2_ interface, but also at the TiO_2_/Pt interface (Figure 4a). While the former governs magnetic modulations, the latter enables dynamic control of longitudinal resistance. As shown in Figure 4b, the resistance of the heterostructure decreases significantly, by up to 46.6 Ω (Δ*R*/*R*_0_ (initial resistance) = 55%), as the voltage is reduced to 0.8 V. This resistance drop is minimal in the low‐density region (between 3.0 and 2.0 V), but becomes pronounced below 2.0 V, mirroring the *M*_s_ trends observed in Figure 3c. These results confirm that both *M*_s_ and resistance variations originate from the electrons accumulation effect. The resistance modulation is primarily attributed to the Pt layer for two key reasons. First, in the Pt (2 nm)/Co (1 nm) bilayer, Pt dominates the overall conduction channel. Second, the band structure of Pt allows for more effective electron doping than Co. Due to the high effective mass of *d*‐electrons, additional electrons in *d*‐band of Co have limited impact on conductivity. However, in Pt, the nearly filled *d*‐band plays a critical role in *s−d* electron scattering, which governs electrical transport [31, 32]. As illustrated in the simplified band structure inset of Figure 4b, electron doping in Pt elevates the Fermi level, reducing scattering and thereby lowering resistance.

To further validate the role of the TiO_2_/Pt interface, we examined resistance modulation across different heterostructures (Figure 4c). Significant resistance changes are observed only when the bottom TiO_2_ layer adjacent to Pt layer is retained. Replacing it with MgO eliminates the modulation, confirming that interface‐charge at the TiO_2_/Pt interface is essential for the resistance control. This conclusion is reinforced by experiments varying the thickness of the bottom TiO_2_ layer (Figure 4d). Thinner TiO_2_ layers exhibit reduced dielectric capacity, weakening interface‐charge storage and diminishing resistance modulation. These findings confirm the bottom TiO_2_/Pt interface as the primary site for resistance tuning. It is worthing noting that the control experiments demonstrate the modulation of magnetic moment is not originated from the resistance change. Figure 4c illustrate that resistance remains unchanged in MgO/Pt/Co/TiO_2_ across the full voltage range. In Figure S14, however, a significant reduction in magnetic moment is observed from 3 to 0.8 V within the same structure, indicating that resistance and magnetic moment modulation originate from different interfaces. Moreover, Figure S15 presents the resistance variations in Si/Pt(x)/TiO_2_ single‐interface structures with different Pt thicknesses. The resistance variation gradually decreases with increasing Pt thickness, confirming the resistance modulation originates from the TiO_2_/Pt interface.

Additionally, the resistance can be continuously modulated using a sequence of ramped voltage pulses, as shown in Figure 4e. Lower voltages (lithiation) promote electrons accumulation and resistance reduction, while enhanced voltages extract out of interface‐charge (de‐lithiation), restoring resistance. Device stability and endurance are demonstrated in Figure S16, where over 90 charge‐discharge cycles show minimal degradation. Importantly, both magnetic and electrical modulations fully recover to their initial states without external power (Figure S17), confirming the reversibility of the non‐faradaic interface‐charge mechanism. It is worth noting that the volatility [33, 34] is particularly relevant for neuromorphic computing, where volatile states and recovery dynamics can be harnessed for short term memory, neuron like responses, and reservoir computing systems. Moreover, volatility is also advantageous for sensing applications [35], as the device naturally resets to its baseline after stimulation, enabling repeated detection of short‑lived signals without external reset.

## 3 Conclusions

We demonstrate multifunctional modulation of magnetism and resistivity in common metals with large amplitude, reversibility, and durability through interface‐charge mechanism. The spatially separated ions and electrons generate an interfacial capacitor in the high voltage range and high‐density electron accumulation in the low voltage range, enabling reversible and giant tuning of magnetic anisotropy, saturation magnetization, and electrical resistance in the Pt/Co bilayer. The reorientation of Co magnetic anisotropy from out of plane to in plane, accompanied by substantial reductions in saturation magnetization (up to 44%) and electric resistivity (up to 55%), are not only pronounced but also highly reversible and stable, underscoring the effectiveness and robustness of the approach. Our findings establish the interface‐charge as a scalable and versatile platform for non‐faradaic control in metallic systems, unlocking new possibilities for electrically reconfigurable spintronic devices, sensors, and neuromorphic computing technologies.

## 4 Experimental Section

### 4.1 Sample Preparation

All films were deposited on thermally oxidized Si (001)/SiO_2_ substrates at room temperature by magnetron sputtering using 8×8 mm^2^ masks. The Co and Pt layers were deposited using direct current (dc) sputtering with Ar gas pressure of 3 mTorr. TiO_2_ and MgO were prepared using radio frequency (rf) sputtering with 6 mTorr Ar. During the sputtering process, the substrate was rotated at 15 rpm to ensure uniform thickness. All deposition rates were calibrated by X‐ray reflectivity.

### 4.2 Electrochemical Measurements

The multilayers were assembled as positive electrodes in an argon‐filled glovebox. The electrolyte was 1.0 m LiPF_6_ in 1:1 w/w dimethyl carbonate (DEC)/ethylene carbonate (EC). A Celgard 2250 film was utilized as the separator (Whatman) and Lithium foil was used as the counter electrode. The galvanostatic charge/discharge (GCD) measurements of the cells were evaluated on a multichannel battery tester (NEWARE CT‐4008) within the voltage range of 0.8–3.0V. CV tests were implemented over the range of 0.8–3.0V on an electrochemical workstation (CHI660E) and a Keithley 2450 source meter. All the electrochemical measurements were carried out at room temperature.

### 4.3 Transport Measurements

The electrical transport characteristics were measured utilizing the Van der Pauw configuration, enabling the simultaneous recording of both longitudinal resistance and Hall resistance. The gate voltage was generated by a Keithley 2450, while the test current was provided through a Keithley 6221. The Hall and longitudinal voltage were recorded via Keithley 2182A. All measurements were conducted with a test current of 0.1 mA at room temperature.

### 4.4 Computational Details

First‐principles calculations were carried out using the Vienna ab initio Simulation Package (VASP), which is based on density functional theory (DFT) [36, 37]. The projector augmented‐wave (PAW) method was utilized to describe the electron‐ion interactions [38, 39], and the exchange correlation was treated by the Perdew‐Burke‐Ernzerhof (PBE) functional within the generalized gradient approximation (GGA) [40]. A kinetic energy cutoff of 450 eV was used in the calculations. The convergence criteria for the total energy and atomic forces were set to 10^−8^ eV and 0.005 eV/Å, respectively. A Γ‐centered Monkhorst‐Pack k‐point grid of 35  ×  35 ×  1was adopted for Brillouin zone sampling. Additionally, to eliminate spurious interactions between adjacent structures, a vacuum space of more than 20 Å was added along the z‐axis. The Co/Pt heterostructure used in our calculation was constructed by stacking four atomic layers of hcp Co (001) onto six atomic layers of fcc Pt (111), where the first two atomic layers of Co follow the fcc pattern of Pt (111). The lattice constant of the heterostructure was fixed at 2.507 Å, corresponding to the lattice constant of bulk hcp Co.

## Author Contributions

**Qiang Cao**: conceptualization, writing – original draft, investigation. **Yingli Li**: data curation, investigation.Zhaohui Li: Writing – review and editing. **Yufeng Tian**: supervision. **Weixin Liu**: software. **Senmiao Liu**: methodology. **Shishen Yan**: conceptualization, validation, writing – review and editing, supervision. **Qiang Li**: supervision, resources. **Shengshi Li**: software. **Xinglong Ye**: data curation. **Zhen Han**: investigation.

## Conflicts of Interest

The authors declare no conflicts of interest.

## Supporting information




**Supporting File**: advs76628‐sup‐0001‐SuppMat.docx.

## Data Availability

The data underlying this study are available from the corresponding author upon reasonable request.
