# Supplementary material for: Interface Charge Induced Multifunctional Manipulations in Metals
Source: Adv Sci (Weinh). 2026 Jul 14;13(56):e76628. doi: 10.1002/advs.76628 (PMC13367100; doi:10.1002/advs.76628)
Supplement: Supplementary file 1 [file ADVS-13-e76628-s001.docx]

Supporting Information

**Interface Charge Induced Multifunctional Manipulations in Metals** Qiang Cao^#^,^1^ Yingli Li^#^,^2^ Senmiao Liu,^1^ Zhen Han,^1^ Weixin Liu,^1^ Zhaohui Li,^3^ Qikun Huang,^1^ Shengshi Li,^1^* Qiang Li,^2^* and Shishen Yan^1^*

^1^Spintronics Institute, University of Jinan, Jinan 250022, China

Email: shishenyan@sdu.edu.cn; sdy_liss@ujn.edu.cn

^2^College of Physics, Weihai Innovation Research Institute, Institute of Materials for Energy and Environment, Qingdao University, Qingdao 266071, China

Email: liqiang@qdu.edu.cn

^3^Department of Electrical and Computer Engineering, National University of Singapore, Singapore 117576, Singapore

Figure S1. Galvanostatic curve over 6 consecutive cycles at a constant current of 10 μA. It exhibits the anticipated capacitive behavior, lacking a distinct electrochemical platform.

 Figure S2. XPS of Co 2*p* peaks (a) and Pt 4*f* peaks (b) measured before and after applying voltage of 2 and 0.8 V. In the full voltage range, the metallic states are unchanged.

 Figure S3. a) A cyclic voltammogram curve with a scan rate of 10 mV s^−1^. Linear relationship between current and sweep rate, derived from the equation $\log i\left( v \right)=\log a+b\log v$, with measurements taken at the cathodic peak (b) and anodic peak (c) over a voltage range of 3 to 2 V.

A pinched cyclic voltammogram (CV) curve in the voltage range of 3 to 2 V suggests a Li^+^ ions insertion process into TiO_2_. No redox peaks can be observable, with a capacitive ratio of 73.6 %. The parameter *b*, which is calculated from the slope of the linear $\log i$ versus $\log v$ plot, provides crucial insights into the kinetics of the electrochemical processes involved. A *b* value of 1 indicates that the behavior is dominated by fast near-surface activities, such as the charging and discharging of electric double-layer capacitors.^[1]^

Figure S4. Magnetization loops measured with magnetic field along out-of-plane and in-plane direction, respectively.

The variation of interface magnetic anisotropy ($K_{i}$) can be evaluated as ${\Delta K}_{i}=\frac{1}{2}{\Delta H}_{k}\mu_{0}M_{S}d_{Co}$, in which ${\Delta H}_{k}=200 mT$. The saturation magnetization is $1.145\times{10}^{6} A/m$; and the Co layer thickness is $1nm=1\times{10}^{-9}m$. Therefore, ${\Delta K}_{i}\approx0.11 mJ m^{-2}$. For a voltage window of $\Delta V=1V$, $\frac{{\Delta K}_{i}}{\Delta V}$ $\approx0.11 mJ V^{-1}m^{-2}$.

.

 Figure S5. The *R*_H_ loops at 3 and 2 V in multilayers where the bottom TiO_2_ layer is replaced by MgO (a) and Pt (b). *R*_H_ loops at 3 and 2 V in multilayers where the top TiO_2_ layer is replaced by MgO (c) and Pt (d). The numbers in brackets represent nanometers.

 Figure S6. Evolution of anomalous Hall resistance (*R*_H_) loops under applied voltage from 3 to 2 V for Co layers with thicknesses of 1 nm (a), 1.4 nm (b), and 1.8 nm (c), respectively.

Figure S7. Atom-resolved MAE in the pristine CoPt multilayer.

Figure S8. Electron distributions in the CoPt system after electron doping. The isosurface value is 0.0017 e/Å^3^.

Figure S9. Variation of the Co-contributed MAE in the CoPt system as a function of electron doping concentration.

Figure S10. Orbital-resolved MAE of Co_2_ and Co_3_ atoms in the undoped CoPt system (a, b) and in the CoPt system doped with 1.2 e/u.c. electrons (c, d).

Figure S11. The PDOS of d orbitals for the Co atoms in the undoped CoPt system (a-d) and in the CoPt system doped with 1.2 e/u.c. electrons (e-h).

Figure S12. a) A cyclic voltammogram curve with a scan rate of 10 mV s^−1^.Linear dependence of the current on the sweep rate measured at the cathodic b) and anodic c) in the voltage range from 2 to 0.8 V.

The CV curve for 2 to 0.8 V exhibits a rectangle, akin to the EDL capacitance, with an extremely high capacitive ratio of 92.7%. This indicates that the Li^+^ ions storage is dominantly capacitive behavior. A *b* value of 1 signifies contributions from capacitive activities as well.

Figure S13. a) Time-resolved modulation of *R*_H_ under an external field of 800 mT by repeatedly applying 10 pulses at 0.8 V and 10 pulses at 2 V. Each pulse lasts 50 ms with a 50 ms interval, followed by a 10 s voltage hold after each pulse series. b) Resistance evolution over time under repeated application of 10 pulses at 0.8 V and 10 pulses at 3 V. The voltage control parameters are identical to those used for *R*_H_ modulations.

 Figure S14. a, b) The *R*_H_ loops under 3 and 0.8 V with the top TiO_2_ layer remaining unchanged. c) The *R*_H_ loops under 3 and 0.8 V with the top TiO_2_ replaced by Pt.

Figure S15. (a) Relative resistance variation for Pt film in Si/Pt(x)/TiO_2_ heterostructures with different Pt thicknesses. The inset illustrates the heterojunction configuration. (b) Interface charge induced resistance evolution over time for various Pt thickness by applying a series of continuous ramped pulse voltages with 15s pulse width.

Figure S16. Chronoamperometry measurement with current of 10 $\mu$A over 90 cycles. The measurement indicates the robustness of our device with minor changes during voltage switching between 3 and 0.8 V.

 Figure S17. a) After being discharged to 0.8 V, the device was set to open circuit and the *R*_H_ loops are continuously monitored for 2 hours. The *R*_H_ loops spontaneously recover to the initial state. b) Temporal evolution of longitudinal resistance by repeatedly applying 3 V and 0.8 V voltage pulses. Each pulse lasts for 15 seconds. Without power supply, the resistance completely recovers in 32 minutes.

**References**

[1]	J. Liu, J. Wang, C. Xu, H. Jiang, C. Li, L. Zhang, J. Lin, Z. X. Shen, *Adv. Sci.* **2017**, 5, 1700322.
